# Focus on Immune Checkpoint Inhibitors-related Intestinal Inflammation: From Pathogenesis to Therapeutical Approach

**DOI:** 10.1093/ibd/izad229

**Published:** 2023-10-06

**Authors:** Angelo Del Gaudio, Federica Di Vincenzo, Valentina Petito, Maria Cristina Giustiniani, Antonio Gasbarrini, Franco Scaldaferri, Loris Riccardo Lopetuso

**Affiliations:** UOS Malattie Infiammatorie Croniche Intestinali, Centro di Malattie dell’Apparato Digerente (CEMAD), Fondazione Policlinico Universitario A. Gemelli IRCCS, Università Cattolica del Sacro Cuore, Roma, 00168, Italy; UOC di Medicina Interna e Gastroenterologia, Dipartimento di Scienze Mediche e Chirurgiche, Fondazione Policlinico Universitario A. Gemelli IRCCS, Università Cattolica del Sacro Cuore, Roma, 00168, Italy; UOS Malattie Infiammatorie Croniche Intestinali, Centro di Malattie dell’Apparato Digerente (CEMAD), Fondazione Policlinico Universitario A. Gemelli IRCCS, Università Cattolica del Sacro Cuore, Roma, 00168, Italy; UOC di Medicina Interna e Gastroenterologia, Dipartimento di Scienze Mediche e Chirurgiche, Fondazione Policlinico Universitario A. Gemelli IRCCS, Università Cattolica del Sacro Cuore, Roma, 00168, Italy; UOS Malattie Infiammatorie Croniche Intestinali, Centro di Malattie dell’Apparato Digerente (CEMAD), Fondazione Policlinico Universitario A. Gemelli IRCCS, Università Cattolica del Sacro Cuore, Roma, 00168, Italy; UOC di Medicina Interna e Gastroenterologia, Dipartimento di Scienze Mediche e Chirurgiche, Fondazione Policlinico Universitario A. Gemelli IRCCS, Università Cattolica del Sacro Cuore, Roma, 00168, Italy; Department of Pathology, Fondazione Policlinico Universitario A. Gemelli IRCCS, Roma, 00168, Italy; UOS Malattie Infiammatorie Croniche Intestinali, Centro di Malattie dell’Apparato Digerente (CEMAD), Fondazione Policlinico Universitario A. Gemelli IRCCS, Università Cattolica del Sacro Cuore, Roma, 00168, Italy; UOC di Medicina Interna e Gastroenterologia, Dipartimento di Scienze Mediche e Chirurgiche, Fondazione Policlinico Universitario A. Gemelli IRCCS, Università Cattolica del Sacro Cuore, Roma, 00168, Italy; UOS Malattie Infiammatorie Croniche Intestinali, Centro di Malattie dell’Apparato Digerente (CEMAD), Fondazione Policlinico Universitario A. Gemelli IRCCS, Università Cattolica del Sacro Cuore, Roma, 00168, Italy; UOC di Medicina Interna e Gastroenterologia, Dipartimento di Scienze Mediche e Chirurgiche, Fondazione Policlinico Universitario A. Gemelli IRCCS, Università Cattolica del Sacro Cuore, Roma, 00168, Italy; UOS Malattie Infiammatorie Croniche Intestinali, Centro di Malattie dell’Apparato Digerente (CEMAD), Fondazione Policlinico Universitario A. Gemelli IRCCS, Università Cattolica del Sacro Cuore, Roma, 00168, Italy; UOC di Medicina Interna e Gastroenterologia, Dipartimento di Scienze Mediche e Chirurgiche, Fondazione Policlinico Universitario A. Gemelli IRCCS, Università Cattolica del Sacro Cuore, Roma, 00168, Italy; Department of Medicine and Ageing Sciences, G. d’Annunzio University of Chieti-Pescara, Chieti, 66100, Italy; Center for Advanced Studies and Technology (CAST), “G. d’Annunzio” University of Chieti-Pescara, Chieti, 66100, Italy

**Keywords:** immune checkpoint inhibitors, colitis, gut microbiota, immuno-related adverse effects, immune system

## Abstract

Recently, antitumor immunotherapies have witnessed a breakthrough with the emergence of immune checkpoint inhibitors (ICIs) including programmed cell death-1 (PD-1), programmed cell death-ligand 1 (PD-L1), and cytotoxic T lymphocyte antigen 4 (CTLA-4) inhibitors. Unfortunately, the use of ICIs has also led to the advent of a novel class of adverse events that differ from those of classic chemotherapeutics and are more reminiscent of autoimmune diseases, the immune-related adverse events (IRAEs). Herein, we performed an insight of the main IRAEs associated with ICIs, focusing on gastroenterological IRAEs and specifically on checkpoint inhibitor colitis, which represents the most widely reported IRAE to date. We comprehensively dissected the current evidence regarding pathogenesis, diagnosis, and management of ICIs-induced colitis, touching upon also on innovative therapies.

## Graphical Abstract

**Graphical Abstract:**
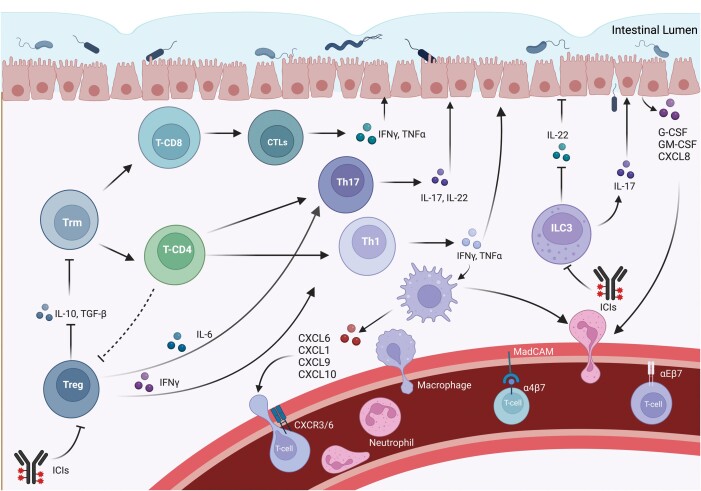

**Key Messages**

Immune checkpoint inhibitors are promising new cancer immunotherapies that have seen a breakthrough recently for the treatment of several cancer types. Herein, we comprehensively analyzed current evidence on IRAEs induced by ICIs in different cancer types, shedding light on new insights. We analyzed the immunological pathways and the role of gut microbiota in the pathogenesis of major IRAEs, particularly ICI-induced colitis. Subsequently, we described signs and symptoms characteristic of colitis onset, as well as the best diagnostic and therapeutic workup for appropriate diagnosis and management, thus providing practical help to clinicians for early recognition and management of this potentially life-threatening adverse event.

## Introduction

The concept of harnessing the immune system in treating malignancies arises from the continuous activity of the immune system, both innate and adaptive, in recognizing and eliminating transformed cells.^1^ However, tumor cells can implement evasion mechanisms, such as reducing the expression of epitopes, forming physical barriers, or interfering with immune checkpoints.^1^ Particularly, immune checkpoints represent an important self-regulating system, which is useful in limiting hyperactivation of the immune system, maintaining homeostasis, and preventing autoimmunity.^2^

The improvement of knowledge in the field of immuno-surveillance and immuno-escape mechanisms has revolutionized cancer treatment, starting from the injection of live bacteria and leading to the development of monoclonal antibodies to inhibit specific targets.^3^

Current strategies involve the blockade of the key immune regulatory “checkpoint” receptors, such as cytotoxic T lymphocyte-associated protein (CTLA)-4, programmed death (PD)-1, and its ligand (PD-L1). Other approaches include the administration of oncolytic viruses, cancer vaccines, cytokines therapies, or application of chimeric antigens receptor cells-T (CAR-T).^3^ However, checkpoint inhibitors (ICIs) are currently the mainstay of immunotherapy. Since the US FDA approval in 2011 of ipilimumab, an anti-CTLA-4 monoclonal antibody, for the treatment of unresectable metastatic melanoma, immuno-checkpoint inhibitors have undergone a modest expansion in terms of development and application.^4^ Indeed, currently approved drugs directed against the PD-1/PDL-1 system include nivolumab, pembrolizumab, atezolizumab, avelumab, durvalumab, and cemiplimab.^4^ These drugs are applied in the treatment of several advanced solid tumors, such as metastatic melanoma, colorectal carcinoma, hepatocellular carcinoma (HCC), lung neoplasms as nonsmall cell lung cancer (NSCLC), malignant pleural mesothelioma, renal cell carcinoma (RCC), and urothelial carcinoma.^5,6^ Furthermore, hematological neoplasms include Hodgkin’s lymphoma and primary mediastinal large B cell lymphoma.^7^ Basically, CTLA-4 and PD-1 are co-inhibitory receptors exposed on the surface of T cells that can downregulate T cell–mediated immune responses; however, tumor cells utilize these inhibitory molecules to induce tumor tolerance. Hence, anti-CTLA-4, anti-PD-1, and anti-PD-L1 can bind these co-inhibitory receptors, reactivating the immune response against tumor cells.^8-10^

In detail, CTLA-4 is an inhibitory receptor expressed by CD8 T lymphocytes and Treg cells, which returns T cells to a resting state in response to inhibitory stimuli.^8^ Specifically, the naïve T lymphocyte requires both antigen stimulation of the T cell receptor and CD28-mediated costimulation to be activated.^8^ The CTLA-4 competes with CD28 for specific ligands presented on antigen-presenting cells (APCs); when activated, it generates a T-cell inhibitory signal, reducing the availability of CD-28 ligands.^11^ Furthermore, PD-1 is a receptor expressed by many activated immune cells such as macrophages, dendritic cells, and B-T-lymphocytes.^9^ Through interaction with the ligands PD-L1 and PD-L2, its activation results in the suppression of the T cell–mediated immune response.^9^ Furthermore, it would appear that PD-1/PD-L1 blocks affect the late proliferation of T cells and, therefore, trigger a more localized immune reaction, unlike blocking CTLA-4, which acting more upstream would result in a more generalized immune response.

Not surprisingly, mice presenting gene deletion of either CTLA-4 or PD-1 develop severe systemic autoimmune diseases.^9^ There is much evidence of the interaction between tumor cells and these regulatory systems; interestingly, a correlation between prognosis and the expression levels of PD-L1 and PD-L2 in several tumor tissues has been found.^12^ Moreover, the interaction between PD-1 expressed by T lymphocytes and PD-L1 expressed by tumor cells is associated with faster tumor growth.^12^

## Immuno-related Adverse Effects

Unfortunately, only a minority of patients undergoing these treatments achieve a lasting response because of emerging resistance phenomena or adverse effects that limit their use, necessitating temporary or permanent discontinuation.^13^The exact pathophysiology underlying these effects is not yet fully understood; however, they probably are a consequence of the pharmacodynamics of these drugs, which interact with immuno-tolerance mechanisms that prevent inappropriate reactions to self-antigens and commensal microorganisms.^13^ Other mechanisms probably involved include dysbiosis-mediated increased exposure of innate immune cells to microbial antigens or epitope spreading that may lead to self-reactivity.^14^ Approximately 85% of patients on therapy with ipilimumab and 75% of those receiving PD-1 axis inhibitors reported immunotherapy-related adverse events (IRAEs).^15^ The toxicity of these therapies seems to be mostly dose-related, and patients on combination therapy show a higher incidence of adverse effects.^15^ Compared with chemotherapy, IRAEs generally have a longer duration and show a mild to moderate severity.^15^ The onset may occur weeks or months after the start of therapy or even after its conclusion. The severity of the manifestations is graded from 1 to 5, on an increasing scale, according to the Common Terminology Criteria for Adverse Events (CTCAE) from the US National Cancer Institute.^15^ Several systems can be affected; the most involved are the surface of barriers, such as the skin, lungs, or gastrointestinal tract; although the endocrine, cardiovascular, ocular, and musculoskeletal systems are also involved.^16^ When the toxicity occurs during the course of therapy with ICIs, the event is considered acute; if it occurs at the end of the treatment, it is considered delayed; and if it persists more than 12 weeks after discontinuation, it is considered chronic.^17^ Endocrinological and rheumatological manifestations tend to chromicise more frequently than other manifestations such as colitis.^17^

## Gastrointestinal Toxicities

Focusing on the gastrointestinal system, the manifestations described vary and may affect the upper and lower digestive tracts, the hepatobiliary system, and the pancreas. Considering the upper digestive tract, nausea, gastritis, duodenitis, and oesophagitis have been observed.^18^

Nausea is a nonspecific symptom, often accompanied by vomiting; it is reported by 12% of patients during PD-(L)1 inhibitor, 19% of those treated with CTLA-4 inhibitors, and 25% when combined.^13^ Nausea and vomiting are generally mild manifestations, especially when they occur in an isolated form, but increase in severity when associated with other conditions such as infections, endocrinopathies, or organ damage; however, only 2% of cases have a grade >3.^19^ Rarely, gastric ulcerations, lymphocytic gastritis, cytomegalovirus-related gastritis, neutrophilic gastritis, haemorrhagic gastritis, and ulcerative esophagitis have been observed.^20^ Upper GI manifestations may appear either 1 to 2 months after the start of therapy or months or years after termination.^19^ Furthermore, although rare, both histological and endoscopic gastric involvement has been found more frequently than duodenal involvement.^19,20^ Interestingly, rare cases of new onset of celiac disease have been described, although its relationship with ICI treatment is not well demonstrated, and it is unclear whether the therapy with ICIs simply acts as a trigger factor that exacerbates an underlying subclinical enteropathy, leading to overt clinical manifestations, or induces celiac disease ex-novo, as it has been previously reported for other immune-related disorders.^21,22^ In the majority of cases described, the gluten-free diet was sufficient to reverse the damage, and there was no therapeutic interruption.^22^

Notably, 2 main clinical phenotypes of small bowel involvement related to ICIs have been identified: the first is characterized by enteropathy with villous atrophy (VA), whether or not related to celiac disease, and the second is characterized by generally severe ulcerative enteritis, sometimes with massive gastrointestinal bleeding or perforation of the small intestine but without any mention of VA.^22,23^

The first group generally involves patients on monotherapy with ICI. In the described cases of villous atrophy of the small intestine, only a minority of patients have positive celiac antibodies, while almost all have increased duodenal intraepithelial lymphocyte counts, mimicking celiac disease. The main histologic discriminator from the celiac disease is the presence of inflammatory activity (defined as neutrophilic infiltrates and/or erosions) always found in ICI-associated duodenitis biopsies but not in celiac disease. It is also noteworthy that rare cases of diffuse ulceration of the duodenum and villar atrophy have been described. The increase in CD8 T cells in the lamina propria and the decrease in the CD4 to CD8 T cells ratio are characteristic of ICI-associated VA, consistent with the mechanism of action of these therapies, which suppress the activity of regulatory T cells.^24^ Usually, the onset of villous atrophy manifests with nonbloody diarrhea and weight loss. In the majority of cases, despite the present of celiac serology, a gluten-free diet is usually started, and clinical symptoms improve with the administration of immunosuppressants and resolve permanently after discontinuation of therapy in almost all cases of VA. Similar to the case with colonic involvement, the use of steroids, in particular, budesonide administered according to the Mayo Clinic open capsule schedule, and infliximab may be useful in treating the side effects of ICI related to small bowel involvement.^22,23,25^

On the other hand, combination therapy with ipilimumab/nivolumab appears to be more common in patients who develop small bowel involvement other than VA, such as ulcers ranging from small aphthous ulcers to diffuse small bowel ulcers with small bowel perforation, while on ICI treatment.

In this group of patients, diarrhea is still the most common symptom, although the clinical picture is generally more severe, in some cases with life-threatening gastrointestinal bleeding or small bowel perforation. Therefore, due to the severity of symptoms, this latter group often requires rescue therapy with infliximab or surgery.^22,23^

Upper gastrointestinal tract toxicity, particularly ICI-associated gastritis, is characterized by less severe inflammation of the lamina propria, mainly because of fewer plasma cells and CD20 B cells within the lamina propria, and demonstrates more intraepithelial lymphocytes, primarily because of significantly higher numbers of intraepithelial CD8 T cells. Moreover, it shows fewer lymphoid aggregates compared with *Helicobacter pylori* gastritis.^26^ It is usually managed according to the severity of the manifestation, suspending, or continuing ICIs treatment and administering PPIs or anti-H2 for milder cases or corticosteroids and antitumor necrosis factor (anti-TNF) alpha therapy for refractory cases.^27^

Considering the hepatobiliary system, cases of hepatitis and occasionally cholangitis and cholecystitis have been reported.^27^ Hepatitis is defined based on the elevation of transaminases alanine aminotransferase (ALT) and aspartate aminotransferase (AST). It appears to occur with an approximately comparable incidence (5%) in patients treated with anti-CTLA-4 and PD-1 axis inhibitors, whereas a higher incidence (19%) is registered with the combination of the 2 drugs.^28^ The severity of damage varies depending on the drug considered; combination therapy and anti-CTLA-4, especially ipilimumab, present the highest risk of severe hepatitis.^29^ The spectrum of manifestations varies from modest transaminase elevation to fulminant liver failure.^29^ The grade of damage and its frequency appears to be greater when these therapies are applied in the treatment of hepatocellular carcinoma than in other malignancies.^29^ The damage pattern is mainly hepatocellular; however, mixed or purely cholestatic patterns have been described.^29^ Management is based on disease severity, starting with corticosteroid therapy and progressing to immunomodulators such as mycfenolate mofetil or tacrolimus for steroid-refractory cases.^30^ Concerning pancreatic involvement, increased amylase or lipase values are relatively frequent, with an incidence ranging from 2% to 8% depending on the therapy used, although pancreatitis is a rare event.^19^ It has recently been named Type 3 autoimmune pancreatitis, but the diagnosis and therapy are poorly understood in the literature.^31^ The diagnosis is based on temporal association with drug use, but sometimes radiological criteria are absent. Cortisones are frequently used for treatment, but their real benefit is still unclear.^31^

## The Lower Digestive Tract: Focusing on Colitis

Due to their frequency, severity, and complex management, adverse effects of the lower intestinal tract deserve special attention. Diarrhea represents a widespread manifestation, occurring in about 11% of patients receiving therapy with PD-1 axis inhibitors, while higher frequencies are recorded with CTLA-4 inhibitors (36%) and with the combination of the 2 drugs (44%).^32^ In such cases, diarrhea is a warning symptom for the possible presence of underlying colitis. Colitis is an inflammatory condition of the colon and represents the main ICIs toxicity in the gastrointestinal setting. The severity of diarrhea and colitis have been classified into 5 stages according to the CTCAE version 5.0 modified by the NCCN Panel, based on the intensity of abdominal pain, the number of evacuations, the presence of blood or mucus in the stool, and the occurrence of complications.^33^Table 1 summarizes this classification.

**Table 1.** Grading system for colitis-diarrhea.^33^

Grade 1	Grade 2	Grade 3	Grade 4
- Increase of 4 bowel movements per day - Slight increase in ostomy output over baseline - No symptoms of colitis (watery diarrhea, cramping, urgency, abdominal pain, blood and mucus in stool, fever, nocturnal bowel movements)	- Increase of 4-6 bowel movements per day - Moderate increase in ostomy output over baseline - Mild/moderate colitis symptoms: - Nocturnal bowel movements	- Increase of 6 bowel movements per day - Severe increase in ostomy output compared with baseline - Severe colitis symptoms: - Hemodynamic instability - Hospitalization indicated	-Same as grade 3, but with: -Other serious/life-threatening complications -Urgent intervention indicated

The incidence of colitis varies depending on the treatment considered, the characteristics of the patient, and the underlying malignancy.^34^ Overall, an incidence of colitis ranging from 3% to 15.5% is reported for patients treated with CTLA-4 inhibitors, 0.7% to 2.6% for patients treated with anti-PD-1/L1 inhibitors, and 0.7% to 12.8% for those on combination therapy.^34^ In particular, the latter strategy and therapy with CTLA-4 inhibitors appear to have a stronger influence on the occurrence of severe colitis (grade 3-4).^28^ Colitis resulting from PD-(L)1 inhibition is characterized by variable onset over the course of treatment and often with a more indolent presentation compared with that resulting from CTLA-4 inhibition.^34^ Uncertain data concern the relationship between the incidence of colitis and the dosage of therapy: in cohorts in which different dosages of pembrolizumab are administered, the incidence of colitis does not seem to differ.^35^ On the other hand, the use of high dosages of ipilimumab, either as monotherapy or in combination with PD(L)1 inhibitors, results in an increased frequency of this adverse event compared with low dosages.^36^

The onset of ICIs colitis can be variable, occurring either during the start of treatment or after the end of it; the drug administered also contributes to this variability. The overall occurrence varies between 0 to 6.3 months.^37^ Regarding the therapy considered, CTLA-4 inhibitors appear to cause a later onset of colitis than PD-1/PD-L1 inhibitors, whereas the combination of the 2 seem to lead to the earliest incidence. Respectively, the median onset is 6 to 7 weeks for CTLA-4 inhibitors, about 1 week to 2 years for PD-1/PD-L1 inhibitors, and a median of 7 weeks (with a range of 0 to 51 weeks) for combination therapy.^38^

## Pathogenesis

**Figure 1.**
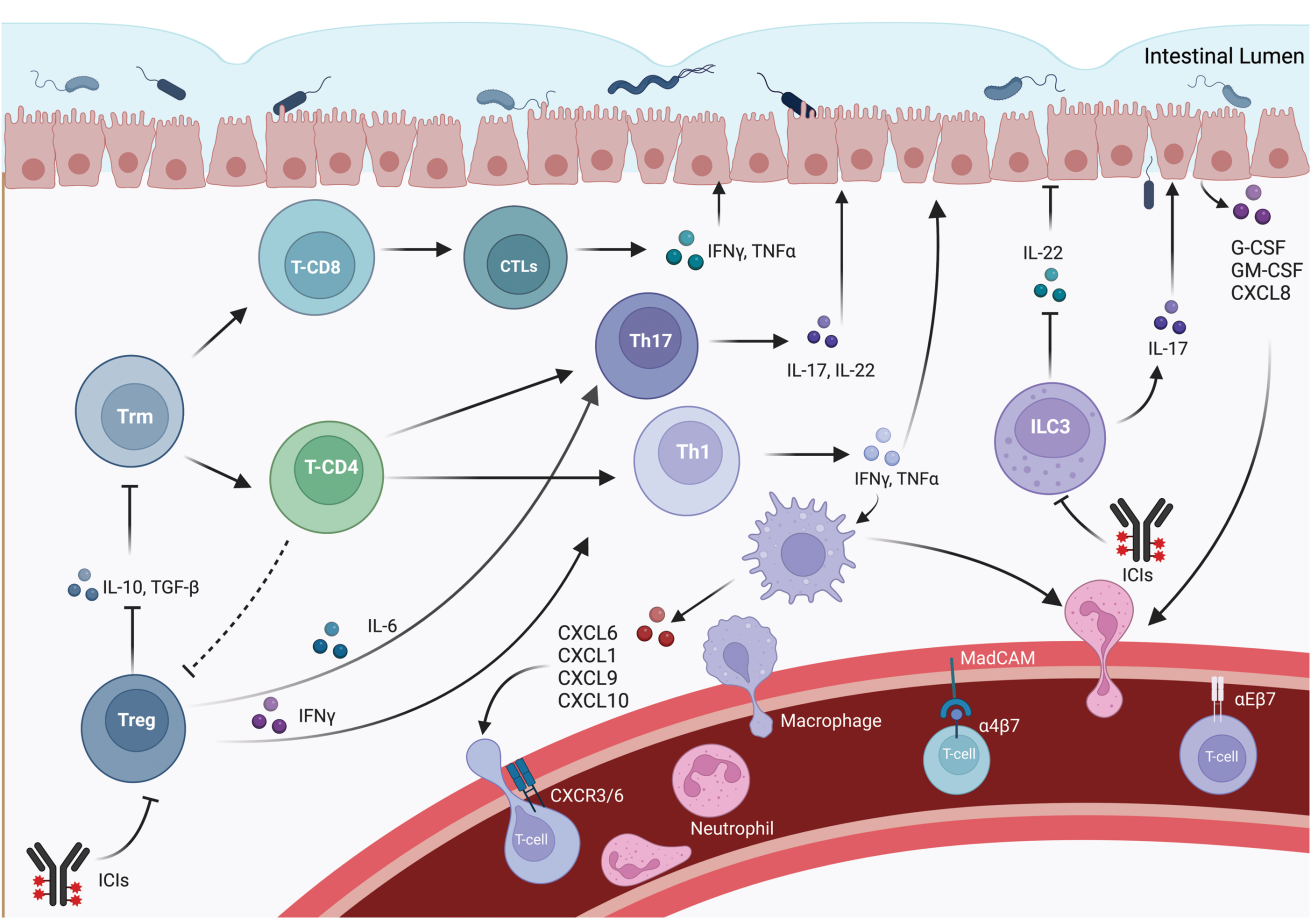
Immunological pathways underlying the pathogenesis of ICIs colitis. Tissue-resident memory T cells (Trm) are recruited and differentiate in cytotoxic effector CD8+ T cells and CD4+ T cells, due to the absence of IL-10 produced by regulatory T cells (Treg). Moreover, Treg usually show gene expression related to an IFNγ -mediated pro-inflammatory Th1-type effect. IL-6 shifts TGF-beta-mediated differentiation of CD4+ naïve cells into Tregs towards Th17 differentiation, even reprogramming Tregs into Th17 cells. The lack of IL-10, enhanced by the production of IL-6 lead to the activation of Th17 cells, which, in turn, produce the proinflammatory IL-17, leading to the development of colitis. Through stimulation of intestinal epithelial cells (IEC) and increased production of G-CSF, GM-CSF, and CXCL-8, IL-17 is also implicated in the recruitment and infiltration of neutrophils and macrophages from systemic circulation. Additionally, lymphocyte recruitment is mediated by the interaction between integrin α4β7, but also αEβ7 on CD8+ lymphocytes and MadCAM on the intestinal endothelium. Macrophages also fulfil a significant role in T-cell recruitment during ICI colitis pathogenesis, mainly through IFN-induced expression of ligands of CXCR3 (CXCL9 and CXCL10) and CXCL-1. The CXCR6 -CXCL16 pair is another crucial couple responsible of T-cell recruitment. Furthermore, the severity of ICI colitis is positively correlated with the number of group 3 innate immune cells (ILC3) that, under ICIs therapy, switch from the production of the anti-inflammatory interleukin-22 (IL-22), which maintains intestinal homeostasis, to IL-17 production. Abbreviations: Trm, Tissue-resident memory T-cell; Treg, Regulatory T cell; T-CD, T cell Cluster of Differentiation; CTLs, Cytotoxic T lymphocytes; Th, T helper cell; ILC3, Type 3 innate lymphoid cell; IFN-γ - Interferon gamma; TNF-α, Tumor necrosis factor-alpha; IL, Interleukin; G-CSF, Granulocyte Colony Stimulating Factor; GM-CSF, Granulocyte Macrophage Colony Stimulating Factor; CXCL, C-X-C-Motif Chemokine Ligand; ICIs, Immune checkpoint inhibitors.

Although the mechanisms underlying immunotherapy toxicity are complex and still not well understood, the pathogenesis of immunotherapy-mediated colitis appears to be related to autoimmune events (Figure 1).^39^ Hyperactivation of T-cell effectors, increased memory T cells, lymphocyte infiltration, cytokine activation, and alteration of the relationship between gut microbiota and the immune system would have a crucial role.^39^ In immunotherapy-mediated colitis, an abundant infiltration of CD4 + T-cells and CD8 + T-cells has often been found, with the first being more prevalent in patients receiving anti-CTLA-4 therapy and the second occurring more commonly in anti-PD-1-induced colitis.^40^ Interestingly, the extent of this elevation appears to be more pronounced in ICI-induced colitis than in IBD.^41^ Particularly, analysis of the T-cell receptors of activated cytotoxic effector CD8 + T cells shows the probable origin of these cells from CD8 + tissue-resident memory T cells (Trm), motivating the early onset of symptoms following the initiation of therapy.^39^ Furthermore, at the tissue level, patients with colitis show a reduction in tissue-resident memory T cells (Trm), in contrast to other patients undergoing ICI without colitis.^42^ Moreover, CTLA-4 is overexpressed in regulatory T cells, which are involved in maintaining immune tolerance. The CTLA-4 inhibitors, by inactivating intratumoral and intestinal CTLA-4 + Treg cells, may promote the activation of effector T cells, such as cytotoxic T- lymphocytes (CTLs), resulting in the development of ICI colitis.^43^ However, in these patients, Treg cells do not tend to be reduced; these cells usually show gene expression related to an interferon (IFN)γ-mediated pro-inflammatory Th1-type effect.^42^ Indeed, increased levels of IFNγ and TNFα, the cytokine TNF-like 1A (TL1A) and its receptor DR3 are commonly found.^44^

Treg cells also secrete interleukin (IL)-10, an important cytokine for inhibiting colonic inflammation. Through deletion of the IL-10 receptor gene (IL-10R) in Treg cells, the development of colitis under the action of Th17 cells is observed in mouse models. These results highlight the indispensable role of IL-10 in retaining the stability of the intestinal environment.^45^ An important pathogenetic role is probably assumed by IL-17, a pro-inflammatory cytokine mainly produced by Th17 cells, which participates in the pathogenesis of various autoimmune diseases and promotes tumor progression. The serum level of IL-17 was shown to be elevated during the course of ICI colitis, while basal IL-17 was correlated with severe diarrhea during immunotherapy.^46^ Through stimulation of intestinal epithelial cells (IECs) and increased production of G-CSF, CXCL-8, and GM-CSF, IL-17 is also implicated in the recruitment and infiltration of neutrophils and macrophages, cells of innate immunity whose role is described later on.^47^ Additionally, lymphocyte recruitment is mediated by the interaction between integrin α4β7 and mucosal addressin cell adhesion molecule-1 (MadCAM) on the intestinal endothelium; expression of both genes coding for the integrin α4β7 receptor is observed in the T cells of these patients.^48^ Furthermore, in CD8 lymphocytes, increased expression of genes coding for integrin αEβ7, which is responsible for the retention of these cells in the gut, was also observed.^39,48^ A monoclonal antibody targeting the β7 integrin chain, shared by both α4β7 and αEβ7 receptors, is in clinical development.^39^ Gene expression analysis of colitis-associated T-cell populations revealed high levels of receptor genes for specific chemokines such as chemokine receptor CXCR6. Particular attention is paid to the CXCR6-CXCL16 pair (the gene encoding the ligand for CXCR6), as they could represent potential targets for the treatment of colitis, especially due to the involvement of this chemokine-receptor pair in cancer cell metastasis.^49^

## The Role of the Innate Immune System

Knowledge about the role of innate immunity in these processes is currently limited; however, its involvement in the regulation of T-cell tolerance and modulation of the T-cell response is well known. Elevated neutrophils, increased myeloid chemo-attractants, and factors associated with T-cell and natural killer (NT-cell) activation (eg, CXCL1, CXCL3, IL8, IL19) are frequently found in patients with colitis, in addition to inflammatory cytokines such as IFN-γ and IL-6.^50^ Particularly, the role of IL-6 has been evaluated in several studies, which report the efficacy of IL-6 blockade on increasing the antitumor efficacy of αCTLA-4 and the positive effects on gastrointestinal toxicity of the combination of IL-6 blockade and antibiotics.^38^ Macrophages also fulfil a significant role in T-cell recruitment during ICI colitis pathogenesis, mainly through IFN-induced expression of ligands of CXCR3 (CXCL9 and CXCL10), the key Th1 cell surface molecule.^51^ Studies have shown that CXCR3-deficient mice do not develop dextran sulphate (DSS)-induced colitis, confirming the influence of this pathway in disease development.^51^ Furthermore, recent research has shown that the severity of ICI colitis is positively correlated with the number of group 3 innate immune cells (ILC3).^46^ These cells are involved in the regulation of immune responses to bacteria and regulate the balance with the microbiota. Interestingly, the PD-1 axis assumes great influence in the maturation of these cells. The main homeostatic cytokine produced by ILC3s is interleukin-22 (IL-22), which maintains intestinal homeostasis; however, ILC3s can also promote IL-17 production.^52^ Furthermore, PD-1 is expressed on T cells following chronic antigenic stimulation; thus, this pathway has been associated with the downregulation of the T cell response towards constantly present and difficult to eliminate antigens.^53^ During anti-PD1 therapy, it could increase the survival of T-cell clones directed in response to chronic infection or normal microflora, which in the absence of treatment would have been controlled by the PD-(L)-1 axis.^53^

## The Role of Microbiota

In ICI colitis development, the impaired interaction between the immune system and the gut microbiota seems to play an important role, and it appears to have implications for the therapeutic response. The presence of dysbiosis has been proposed as one of the triggering mechanisms of colitis, as microbial-derived products can activate innate immunity and result in activating self-reactive immune cells.^54^ Immunotherapy can lead to alterations in the intestinal barrier by stimulating apoptosis of intestinal cells via intraepithelial lymphocytes (IELs).^55^ In addition, some species appear to be involved in triggering the inflammatory process, while others are involved in continuing it. The abundance of *Faecalibacterium praustnitzy* has been associated with increased cytotoxic T lymphocyte proliferation and recruitment of T-reg and α4β7, cells in the gut and the tumor microenvironment, increasing the efficacy of therapy; however, it appears to lead to the onset of colitis.^56^ In contrast, increased *Bacteroides fragilis*, due to its anti-inflammatory activity, is considered a protective factor against ICI colitis.^57^ In particular, CTLA-4 takes part in an anti-inflammatory pathway in which *B. Fragilis* increases IL-10 levels by reducing inflammation.^57^ The role of the microbiota is also supported by identifying microbiological stool patterns with predictive value on the risk of colitis in patients with melanoma.^56^ Notably, during anti-CTLA-4 treatment, several microbiological profiles have been associated with both increased efficacy of therapy and increased risk of colitis, such as enrichment in Firmicutes and scarcity in Bacteroides in subjects who develop colitis and, conversely, the abundance of members of the Bacteroidetes phylum in colitis-resistant patients.^56,57^

## Risk Factors

As previously reported, some authors suggest intestinal dysbiosis and preexisting, even silent, inflammatory bowel activity as possible “primum movens” of colitis.^50,54^ Consequently in mouse models, immune manipulation, including infections and fecal transplantation with a dysbiotic microbiome, confer susceptibility to intestinal toxicities related to ICI therapy.^50^ In agreement, studies evaluating the course of these adverse events in IBD patients indicate an increased risk of relapses and an increase in severe manifestations.^58^

Recently, it has been reported that concomitant therapies probably influence colitis development, indeed the use of nonsteroidal anti-inflammatory drugs seems to increase this risk; in contrast, administration of vitamin D apparently reduces it.^59^ There are uncertain and conflicting data on the influence of a previous therapy-related adverse event on the probability of relapse with a second check-point inhibitor.^60^ Furthermore, increased basal levels of soluble CTLA-4 (sCTLA-4) have been associated with an increased risk of gastrointestinal toxicity, but its role remains to be elucidated in the future.^61^

On the genetic side, human leukocyte antigen (HLA) haplotypes and polymorphisms in immunoregulatory genes such as CTLA-4 and CTLA-1 have been identified, with an important correlation with the development of ICI colitis, in particular HLA-DQB1*03:01.^62^ In addition to the already discussed treatment-related differences in incidence, the type of neoplasm would also appear to influence risk, including melanoma patients; and stage 3 neoplasms seem to have a higher incidence of colitis than stage 4.^63^ Interestingly, some studies have found an association between ICI colitis and pneumococcal vaccine in the 3 months prior to ICI.^63^ In contrast, the sex and age of patients did not seem to create differences in incidence, whereas Caucasian ethnicity was associated with a higher risk.^63^

## Clinical Manifestations and Diagnosis

Diarrhea represents the main symptom of colitis, often associated with blood; although the absence of blood does not exclude this condition.^33^ Additionally, it may be accompanied by abdominal pain, fever, vomiting, and nausea. Rarely, colic perforation has been described, especially in late diagnosis cases.^64^ To facilitate early diagnosis, patient education is crucial. Indeed, it is important to determine the patient’s basic bowel habits before starting immunotherapy.^33^ For patients with grade-1 diarrhea, laboratory and stool tests should be started to rule out a possible infectious etiology.^33^

At the laboratory level, there are no specific characteristics, but an increase in inflammatory indices, such as C-reactive protein (CRP) can be observed.^65^ Other changes correlated with disease progression are increased creatinine, low albumin levels, and anemia.^66^ Increased levels of eosinophils and serum leucine-rich α 2- glycoprotein (LRG) have also been found; according to the same authors, they may be useful to distinguish from other types of colitis, such as Cytomegalovirus (CMV) colitis.^67^ Fecal calprotectin represents an important marker, useful both as a predictor of disease activity and as a noninvasive biomarker to predict endoscopic and histologic remission, reducing the use of invasive endoscopic examinations.^68^ Furthermore, fecal lactoferrin can also be used to guide the prioritization of endoscopy, as lactoferrin levels were strongly correlated with inflammation observed by endoscopy (sensitivity of 70%) and even more strongly correlated with inflammation detected by histological evaluation of endoscopic biopsy specimens (sensitivity of 90%).^69^

For patients presenting with grade 2 or higher diarrhea/colitis, radiology and gastrointestinal consultation should be considered for further endoscopic evaluation,. Computed tomography (CT), and magnetic resonance imaging (MRI) are the main imaging tests used for colitis, with a positive predictive value for CT close to 90% but a lower negative predictive value (40%-60%).^70^ Furthermore, the correlation with endoscopic findings is quite low.^69^ Frequently found findings are colic distention, bowel wall thickening, and mesenteric vascular engorgement.^71^ Patterns of abnormalities can be grouped into pancolitis and segmental colitis associated with diverticulosis (SCAD) enterocolitis.^71^ In addition, radiological examinations are very useful for the diagnosis of complications such as perforation or toxic colitis.^71^

## Endoscopic and Pathological Findings

Endoscopic evaluation assumes great relevance, as it allows biopsies to be taken in addition to the extent of the disease.^69^ However, performing endoscopy in the absence of symptoms does not add any prognostic information regarding the development of colitis; on the contrary, early evaluation of symptomatic patients appears to be correlated with a shorter duration of colitis and a shorter duration of steroid therapy.^69^ Furthermore, there is no correlation between the clinical symptoms reported by the patients and the endoscopic findings.^72^

**Figure 2.**
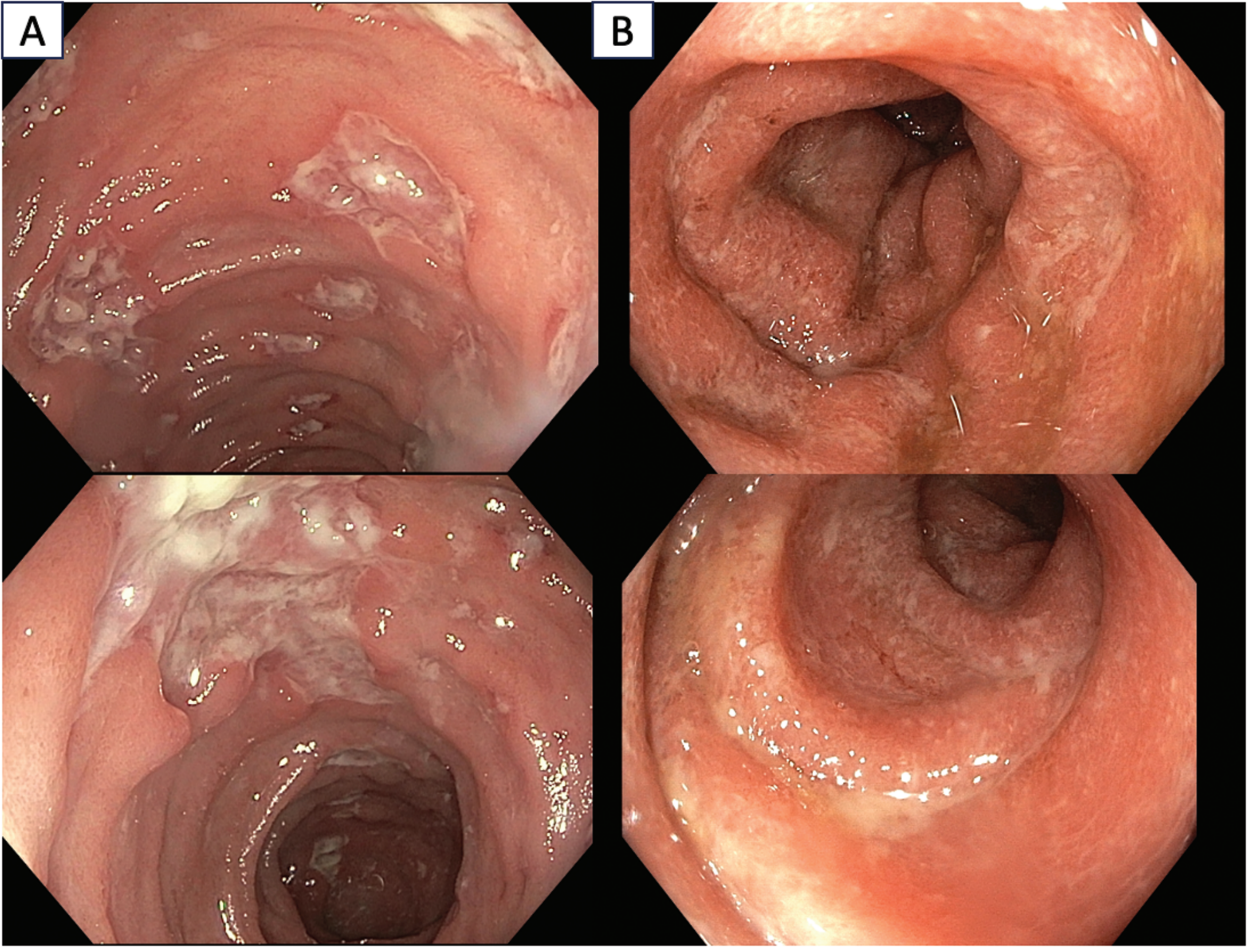
Endoscopic patterns of ICIs colitis. In panel A, Crohn’s like pattern is observed, characterized by oedematous, hyperaemic mucosa with extensive ulcerations, some with a fibrinous base, and pseudo-polyps. In panel B, there is a UC like pattern; the mucosa appears hyperaemic in places with isolated erosions, it is edematous, with disappearance of the submucosal vascular pattern.

Considering the extent of disease, the most frequently reported patterns are left-sided colitis (31%-43%), pancolitis (involvement of ≥3 segments; 23%-40%), and ileitis (11%-14%).^73^ Notably, the occurrence of pancolitis has been identified as a predictor of steroid-refractory colitis.^74^ The detection frequency of ulcerations and nonulcerative inflammation such as edema, erythema, and erosions appear to be similar.^75^ The involvement in most cases is diffuse, resembling ulcerative colitis; however, in some cases patchy and segmental involvement is found, reminiscent of Crohn’s disease.^76^ Examples of endoscopic patterns are shown in Figure 2. Currently, there is no specific scoring system for immunotherapy colitis; however, the Mayo Ulcerative Colitis Score or the Simple Endoscopic Score (SES) for Crohn’s disease are often used.^73^ Abu-Sbeih et al proposed a classification of endoscopic findings into high or low risk patterns of response to steroids.^69^ To the first group belong ulcerations larger than 1 cm and/or deeper than 2 mm or colitis, extending to the splenic flexure of the colon. High-risk lesions have been associated with a higher number of disease recurrences and a greater need for up-grading therapy with biological drugs.^39^

Histologically, the most frequent findings are similar to those of active colitis.^77^ The presence of cryptitis, intraepithelial neutrophil lymphocytes, mucosal ulcerations, crypt abscesses, and apoptosis are common features in ICI colitis.^37,74^ However, these findings seem to be less correlated with the degree of diarrhea.^37^ Another frequently encountered pattern is chronic active colitis; it is characterized by basal plasmacytosis and lymphoplasmacytic infiltration in the lamina propria, metaplasia of Paneth or pseudopyloric cells, and structural distortion of crypts.^78^

**Figure 3.**
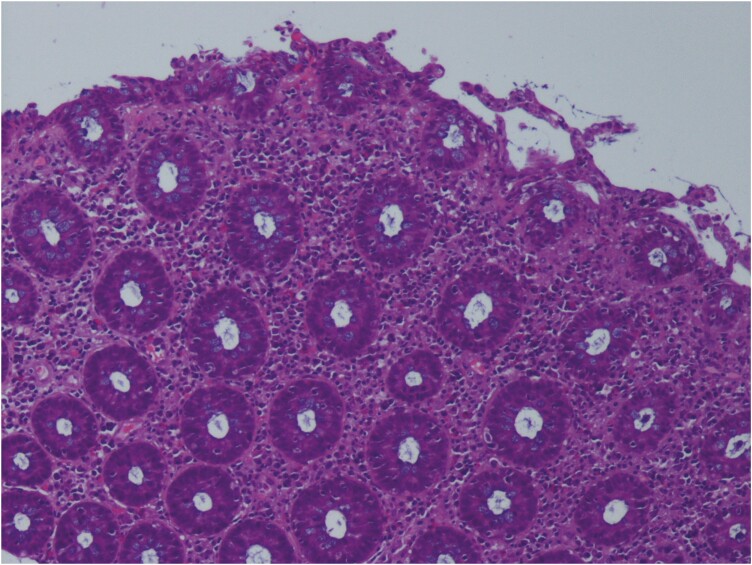
Histological features of ICIs colitis. Colonic section in which preserved architecture, moderate lympho-plasma cellular and neutrophilic granulocytic infiltrate of the lamina propria and areas of cryptitis are observable.

In addition, these features may appear simultaneously with active colitis or develop gradually thereafter, and overlapping patterns may exist in the microscopic presentation of ICI colitis.^79^ In approximately 10%-12% of cases, microscopic colitis (ie, lymphocytic colitis [LC]) and collagenous colitis (CC) are observed.^79^ According to some authors, this condition is associated with a more aggressive disease, with more difficulties in treatment than microscopic colitis developed in the absence of ICIs.^80^Figure 3 shows a histological examination of ICIs colitis.

Rarely, patterns of ischemic colitis, increased apoptosis, and nonspecific inflammatory reactive changes have been described.^79^ Interestingly, Isidro et al reported medication-specific histological changes; in their study, patients on ipilimumab therapy were more likely to show a pattern of diffuse active colitis without chronic features.^81^ Lymphocytic colitis and CC were more common in patients treated with nivolumab and pembrolizumab, while chronic active colitis was more frequent in long-term treatment with nivolumab.^81^ Surprisingly, although nivolumab and pembrolizumab belong to the same drug class, they may be associated with different histological pictures. Notably, there is currently no single criterion that confirms the diagnosis, as none of the histological features are pathognomonic, and there is a wide range of pathological findings that can be observed. Rather, histopathology must be interpreted in the appropriate clinical context, excluding other potential aetiologies.^81^

## Differential Diagnosis

In the diagnostic workup of diarrhea and colitis, given their frequency, it is necessary to exclude infections, whether viral, bacterial, or parasitic.^33^ Patients undergoing ICI therapy have an increased risk of infections, thus it is important to perform microbiological studies and/or stool culture.^33^ Often these conditions can be difficult to distinguish clinically, endoscopically, and histologically from ICI colitis; however, some specific features caused by pathogens can be recognized, helping the distinction.^82^ Cytomegalovirus is an opportunistic infection-causing agent capable of causing ICI colitis–like findings or complicating already present colitis. Indeed, it has been associated with refractoriness of steroid response in ICI colitis.^83^ Diagnosis is usually performed by identifying the typical large cells with basophilic cytoplasm and pathognomonic large, oval, eosinophilic intranuclear inclusions (owl-eye inclusions), or through the application of immunohistochemistry.^84^ Additionally, *Clostridium difficile* infections (CDIs) have been documented in patients undergoing ICI, and CDIs superimposed on ICI-induced colitis have also been described.^85^ Furthermore, the use of proton pump inhibitors (PPIs) and antibiotics during the administration of ICI has been associated with an increased risk of CDI.^85^ Besides infections, many therapies can lead to gastrointestinal toxicity. Patients undergoing immunotherapy may be taking or have previously been treated with chemotherapy or others such as nonsteroidal anti-inflammatory drugs (NSAIDs).^86^ These therapies may lead to conditions that absolutely mimic ICI colitis, such as mycophenolate mofetil damage, which can cause erosions and colic ulcers, with histological findings of cryptitis, cryptic abscesses, or enterocyte apoptosis.^87^ A complete analysis of the drug history and temporal association with the symptoms is essential in these cases. However, some drugs produce characteristic alterations that may be useful in the differential diagnosis. Taxanes, for instance, may deter ring mitotic figures in the proliferative compartment of the intestinal mucosa due to their effect of reducing depolymerization.^88^ Moreover, NSAIDs, in addition to upper gastrointestinal tract damage, can cause lymphocytic colitis and collagenous colitis; however, both are usually patchier and less pronounced in NSAIDs-induced colitis.^87^

In addition, although they may resemble each other, ICI colitis and IBD represent 2 distinct entities. It may be arduous to distinguish disease reactivation from superimposed ICI colitis in an IBD patient undergoing ICI therapy. However, histologically, there are distinctive features that can aid diagnosis, such as the presence of crypt apoptosis, which would favor ICI-induced colitis, as apoptosis is unusual in IBD.^74^ In contrast, noncaseating sarcoidal granulomas and transmural involvement would favor CD, while basal lymphoplasmacytosis would favor both UC and CD.^74^ Furthermore, a higher number of B-lineage cells was observed in UC compared with the anti-CTLA-4-induced colitis.^89^ Clinically, IBD presents an insidious onset, whereas ICI colitis often shows a rapid onset of symptoms after initiation of therapy; moreover, the appearance of lesions such as fistulas may suggest the diagnosis of CD; multiple different organ involvement would favor ICI-induced colitis.^51^

A differential diagnosis also should be made between ICI-induced colitis and other types of ICI-induced enteropathies. However, as explained previously, enteropathies with villar atrophy usually present with nonbloody diarrhea, fatigue, and weight loss, whereas enteropathies with small bowel involvement other than VA commonly present with extensive diarrhea, with the exception of rare cases of severe life-threatening gastrointestinal bleeding or even peritonitis due to small bowel perforation.^22,23^ Therefore, in most cases it is feasible to distinguish enteropathy from ICI-induced colitis already on the basis of the characteristics of the diarrhea. In case of diagnostic doubt, it is still useful to perform upper and lower endoscopy with intestinal biopsies to identify the segment of the intestine involved by the disease and the type of damage.

In summary, the exclusion of infections, investigation of medication history, and their temporary correlation with symptoms, evaluation of multiple organ involvement, and analysis of specific histological features represent absolutely necessary information for a correct differential diagnosis.

## Therapeutic Approaches

### General Considerations

In patients receiving ICI therapy, the occurrence of diarrhea or colitis requires appropriate diagnostic investigation and treatment. Although there is variability in therapeutic practices, management is usually based on the CTCAE classification system. However, it may not be a reliable predictor of the severity of ICI colitis, and as discussed, other diagnostic instruments must be applied.^63^ Therapeutic strategies are mainly based on the use of corticosteroids (CS) and biological drugs such as infliximab (IFX) or vedolizumab (VED) for steroid-refractory CIC patients.^33,90^ Unfortunately, in approximately 1% to 1.5% cases, ICI colitis may complicate with colic perforation, and prompt surgery is necessary.^91^ In these cases, emergency colectomy with subtotal colectomy is the preferred treatment due to the frequently large extent of inflammation.^76^ In general, according to the main guidelines, ICI therapy should be discontinued if the severity of symptoms is greater than grade 2, while for grade 3 to 4 diarrhea/colitis, discontinuation of ICIs should be immediate and permanent.^92^

### Corticosteroids

Corticosteroids, with their inhibiting properties on innate and adaptive immunity, represent the first line of therapy in ICI Colitis, especially from grade 2 upwards.^33^ They also appear to enhance the expression of PD-1 on CD4 and CD8 lymphocytes, reducing the activity of these cells.^93^ However, prolonged and high-dose administration of CS increases the risk of complications such as impaired glucose tolerance, infections, and altered bone metabolism.^94^ Furthermore, the impact of CS activity on reducing the anticancer effects of ICI is still unclear. For these reasons, exposure to CS should be minimized, and therapy should be reduced over 4 to 6 weeks when symptoms improve.^33^ Regarding grade 1 diarrhea/colitis, corticosteroid therapy may be considered in cases with symptoms persisting for more than 2 weeks. In particular, budesonide should be considered initially at a dose of 9 mg/daily for at a minimum of 4 weeks and then tapered over the subsequent 4 to 6 weeks, whereas prednisone may be restricted for patients who are unresponsive to budesonide.^95^ Unfortunately to date, only older formulations of budesonide have been used in case reports and clinical trials of ICI-induced colitis; hence, no data are available on the efficacy of new formulations such as budesonide MMX, which may show particular efficacy in UC-like forms. Indeed, budesonide MMX is a new formulation that, using Multi-Matrix System (MMX) technology, facilitates the release of high concentrations of active drug into the colon. Previous clinical studies have shown that budesonide MMX at a dose of 9 mg/day for 8 weeks is effective and safe in inducing clinical and endoscopic remission in patients with mild to moderate UC who have had an inadequate response or have been intolerant to conventional first-line therapy with topical and oral 5-aminosalicylic acid.^96^ Therefore, it is conceivable that budesonide MMX may also represent a promising treatment for ICI-associated colitis in the UC-like form; however, clinical trials are needed to confirm this hypothesis.

Considering grade 2 cases, the NCCN guidelines recommend administering prednisone/methylprednisolone (1-2 mg/kg/d) with the goal of escalating to 5/10 mg a week after 4 to 6 weeks in case of response.^33^ If there is no improvement within 2 to 3 days, supplementing with biological drugs should be introduced.^97^ For grades higher than 2, hospitalization should be considered in order to assess the electrolyte balance and administer intravenous methylprednisolone 1 to 2 mg/kg/d; although in case of nonresponse, a therapeutic upgrade with biological drugs is strongly recommended^33^

Altogether, approximately 30% to 60% of patients with diarrhea/colitis are refractory to CS, showing no response to high dose within 72 hours of onset or only a partial response after 1 week.^74^

### Biological Therapy

In conditions of steroid refractoriness, and in patients presenting with relapses during steroid tapering or after finishing the steroid course, additional immunosuppressants should be considered.^33,98^ Notably, the incidence of recurrence has been estimated at approximately 44% for CTLA-4 inhibitors and 34% for PD-1/PD-L1 inhibitors, whereas colonic ulceration, pancolitis, and a high Mayo score were identified as predictive factors related to the utilization of secondary immunosuppression.^39^ Furthermore, biopsy samples from patients who required infliximab showed higher CD68 scores and CD8/FoxP3 ratios than those who responded to steroids.^99^ Biologics usually work rapidly, with visible responses in less than a week.^39^ Complete resolution of symptoms may occur after a single infusion of IFX or VDZ, although the relapse rate may be lower in patients receiving 3 infusions compared with 1 or 2.^100^

### Infliximab

Administration of IFX in the treatment of refractory colitis has shown high efficacy in several studies; it is recommended by various societies guidelines such as the ASCO and NCCN.^33,101^ Infliximab is a monoclonal antibody directed against TNF alpha, already in use for IBD and in various rheumatological diseases. Its remission rate in ICI colitis refractory to CS has been estimated to be around 54% to 71.4%.^98^ It is usually administered at a dose of 5 mg/kg/dose intravenously, as in IBD, with a generally rapid response within a few days.^98^

The duration of IFX treatment is still uncertain: about 70% of patients improve with a single administration; however, a subsequent administration 2 weeks later is sometimes necessary.^102^ In some studies, the use of 3 doses (at weeks 0, 2, and 6) appears to reduce the risk of recurrence and increase the probability of endoscopic/histological remission.^100^ Notably, from IBD literature comes the evidence that episodic exposure to infliximab carries a higher risk of anti-infliximab antibodies development compared with continuous therapy, as well as an increased risk of severe acute infusion reactions. Consequently, discontinuation and therapy resumption could also create the same complications in ICI colitis.^103^

Interestingly, some factors such as histological crypt abscesses, early onset of colitis (within 4 weeks after the start of therapy), and preexisting autoimmune disease have been identified as IFX resistance factors.^104^

Furthermore, in comparison to steroids, shorter time of symptoms resolution and hospitalization were reported in patients who received early IFX after CS (within 10 days after colitis onset) compared with patients who received the drug later, suggesting the relevance of early introduction of biological therapy.^100^ In addition, prolonged steroid therapy appears to increase infection risk compared with early introduction of IFX, an issue with high emphasis on the comorbid patient population with malignancies.^105^ However, there are no prospective studies investigating the efficacy of biologics as first-line therapy, although the ASCO guidelines suggest individualized treatment decisions in this setting.^77^ Regarding safety, there are few studies yet, but rare adverse effects have been reported during IFX therapy, such as a rare type of hepatitis, in which case other drugs belonging to the anti-TNF alpha class could be a valid alternative.^106^ Furthermore, the effect of IFX on tumor growth is controversial. Some authors have described good control of neoplasia and colitis with the combination of IFX and ICI.^44^ Others have reported a low impact of IFX therapy on survival and an enhancement of the antitumor effect of ICI by IFX. Indeed, it promotes the cytotoxic T-cell (CTL) activity, inhibits regulatory T-cells (Treg), and also seems to be significantly associated with reduced resistance to PD-1 inhibitors.^44^

In contrast, in other studies of patients treated with IFX showed a reduced overall survival rate compared with patients treated with CS, and long-term use of TNF-α inhibitors seems to be involved in inhibiting the differentiation of CD8+ naïve T cells into CTL by depleting antitumor CTL cells.^107^ For these reasons, IFX administration probably should be discontinued once remission is achieved.

### Vedolizumab

Vedolizumab (VED) is an IgG1 monoclonal antibody that inhibits the entry of activated T cells into the intestine by interacting with the α4β7 integrin expressed by these cells, preventing interaction with the mucosa-directed cell adhesion molecule-1 (MAdCAM-1).^108^ It is a recommended therapy in ASCO and NCCN guidelines, alongside IFX.^19,33^ However, although fewer data are available for VED compared with IFX, it has been reported to be more effective when administered in biologic-free patients (95%) compared with those who had already been treated with IFX.^109^

In addition, this therapy has also shown effectiveness in ICI microscopic colitis.^109^ Vedolizumab is usually injected intravenously with the same schedule as in IBD (at weeks 0, 2, and 6 and every 8 weeks until improvement of symptoms).^108^ There are no definite data yet on the duration of therapy, but a lower risk of relapse and an increased likelihood of endoscopic/histological remission with 3 doses has been reported.^109^ Selective intestinal activity could assume particular relevance in limiting systemic immunosuppression without altering the antitumor response to therapy and without enhancing tumor progression in patients with lymph node involvement.^110^ However, it should be used with caution in patients with gastrointestinal tract tumors or gastrointestinal tract infection. In a retrospective study, Abu-Sbeih et al reported a lower relapse rate in patients receiving vedolizumab compared with infliximab. However, there are currently no studies directly comparing the efficacy of this therapy vs IFX, which would assist in choosing between the 2.

## Other Therapies

Besides those discussed, other therapies have also been applied for the treatment of ICI colitis, such as calcineurin inhibitors (CNIs; tacrolimus and cyclosporine), tocilizumab, and mycophenolate mofetil (MMF).^111^ Tacrolimus has been recommended by the British Society of Gastroenterology (BSG) and the European Society for Medical Oncology (ESMO) for ICI colitis, thanks to its activity of suppression cytokine release such as IL-2, TNF-α, and IFN-γ, inhibiting T-cell activation.^112^

Considering the role of IL-6 in the pathogenesis of colitis and the promotion of tumor progress and metastasis, tocilizumab, an anti-IL-6 receptor antibody, has been investigated as an alternative in the management of ICI colitis.^113^ Interleukin-6 inhibition could obtain both tumor suppression and control of the side effects of therapy. However, except for case reports, there are still few data on the administration of this drug in this context. Notably, an increased risk of intestinal perforation, especially in patients with gastrointestinal ulcers on prolonged concurred CS therapy, has been reported in clinical studies in rheumatoid arthritis (RA) patients.^114^ Therefore, it should be administered with prudence in this category of patients.

Considering MMF, it has been reported that the adjunction of this drug to steroid may be helpful in reducing the duration of treatment.^111^ Mycophenolate mofetil manifests immunosuppressive effects by inhibiting T- and B-lymphocyte replication.^111^ In a study of 11 patients administered MMF, 7 did not develop subsequent colitis flare-ups during CS tapering.^111^ In addition, there are also other potential biologics for ICI-related refractory colitis, such as the anti-IL-1 blockade (anakinra), the anti-IL-17 blockade (ixekizumab), or the blockade of IL-12-23 (ustekinumab), which similarly to the previous drug reported, attempt to target the drivers of the pathophysiological process of IRAEs.^115^

Moreover, small molecules could also have a role in this setting. Indeed, Janus kinase (JAK) inhibitors (tofacitinib) have also shown efficacy in patients with ICI colitis.^116^ However, those reported data are mostly derived from case reports or case series, so further studies are needed to assess the usefulness of these drugs in ICI colitis.

## Fecal Microbiota Transplantation

As discussed, the gut microbiota appears to play a relevant role in the pathogenesis of ICI colitis. In fact, the presence of dysbiosis has been proposed as one of the triggering mechanisms of colitis, and several gut microbiomes are associated with the induction or alleviation of ICI colitis.^57^ For these reasons, fecal transplantation has been proposed as a therapeutic approach for patients with severe or refractory ICI. There are already case series in the literature that have demonstrated a clinical and endoscopic response to fecal transplantation.^117^ This method uses the feces of healthy donors and aims to contribute to the reconstitution of the gut microbiome, leading to immunological changes such as an increase in regulatory T-cells within the colonic mucosa.^117^

Wang et al described the cases of 2 patients with therapy-refractory ICH colitis undergoing fecal microbiota transplantation (FMT).^117^ These patients, in addition to developing complete resolution after 1 and 2 procedures respectively, presented a substantial reduction in CD8+ T-cell density with a concomitant increase in CD4+ FoxP3+ T-cells after transplantation, offering the authors speculation on the potential mechanism by which FMT would reduce ICI-associated toxicity. Despite the promising results, research is still needed to determine the type of microbiota, frequency of FMT, and any risks associated with the method.^116^

## Conclusion

In this narrative review, we comprehensively collected and analyzed the current evidence on IRAEs associated with ICI therapy across cancer types, shedding light on novel, important insights and confirming previous observations about IRAEs. We showed that different tumor types may have different IRAE patterns when treated with the same ICI, while different ICIs are associated with the development of IRAEs with different frequencies. We further investigated the immunological pathways and the role of the gut microbiota currently implicated in the pathogenesis of major IRAEs and, particularly, in the development of ICIs-induced colitis. However, a more thorough understanding of such mechanisms of IRAEs is needed in order to allow the identification of biomarkers that can predict the occurrence of toxicity in patients or predict those who have more severe IRAEs that are unlikely to respond to corticosteroids. Subsequently, focusing on colitis, we showed the main signs and symptoms characteristic of the onset of ICIs-induced colitis, followed by the best diagnostic and therapeutic workup for an early appropriate diagnosis and management of colitis. Considering the number of ongoing clinical trials with ICIs in cancer, there will be a considerable increase in the volume of new data that will require continuous monitoring to further improve our understanding of the risks and benefits of these therapies.
